# Costs and long-term outcomes following pulmonary vein isolation for atrial fibrillation in elderly patients using second-generation cryoballoon vs. open-irrigated radiofrequency in China

**DOI:** 10.1007/s10840-019-00697-7

**Published:** 2019-12-31

**Authors:** Chao-feng Chen, Mei-jun Liu, Chao-lun Jin, Xiao-fei Gao, Xiao-hua Liu, Yi-zhou Xu

**Affiliations:** Department of Cardiology, Affiliated Hangzhou First People’s Hospital, Zhejiang University School of Medicine, #261 Huansha Road, Hangzhou, 310000 Zhejiang Province China

**Keywords:** Atrial fibrillation, Elderly patients, Second-generation cryoballoon ablation, Radiofrequency ablation, Costs and long-term outcomes

## Abstract

**Purpose:**

Limited comparative data are available regarding catheter ablation (CA) of atrial fibrillation (AF) using second-generation cryoballoon (CB-2) vs. radiofrequency (RF) ablation in elderly patients (> 75 years old). The present study aimed to compare the costs and clinical outcomes in elderly patients using these two strategies.

**Methods:**

Elderly patients with symptomatic drug-refractory paroxysmal/short-lasting persistent AF were included in the study. Pulmonary vein isolation (PVI) was performed in all patients.

**Results:**

A total of 324 elderly patients were included (RF,176; CB-2,148) from September 2016 to April 2019. The CB-2 was associated with shorter procedure time and left atrial dwell time (112.9 ± 11.1 vs. 135.1 ± 9.9 min, *P* < 0.001; 53.7 ± 8.9 vs. 65.1.9 ± 9.0 min, *P* < 0.001) but marked fluoroscopy utilization (22.1 ± 3.3 vs. 18.5 ± 3.6 min, *P* < 0.001). Complications occurred in 3.3% (CB-2) and 6.2% (RF) of patients with no significant different (*p* = 0.307). The length of stay after ablation was shorter, but the costs were higher in the CB-2 group (1.94 vs. 2.53 days, *P* < 0.001 and 91,132.6 ± 3723.5 vs. 81,149.4 ± 6824.1 CNY, *P* < 0.001) compared to the RF group. Additionally, the rate of early recurrence of atrial arrhythmia (ERAA) was lower in the CB-2 group (14.2 vs. 23.3%, *P* = 0.047), but the long-term success rate was similar between two groups.

**Conclusions:**

CB-2 is associated with shorter procedure time, left atrial dwell time, and length of stay after ablation, as well as lower ERAA, but its costs and fluoroscopy time are greater than the RF group. Moreover, the rate of complications and long-term success is similar between the two groups.

## Introduction

Atrial fibrillation (AF) is the most common cardiac arrhythmia and affects approximately 10 million individuals in China [1]. The incidence and prevalence of AF increase with age, and it has a striking impact on the morbidity and mortality in older age groups (> 75 years old) [2, 3]. Catheter ablation (CA) is an effective treatment for AF in younger patients; also, some recent studies showed that CA is safe and effective in elderly patients [2, 4–6]. A large number of studies and meta-analyses showed comparable clinical benefits between second-generation cryoballoon (CB-2) and radiofrequency (RF) ablation, but most of them exclude elderly patients [7–9]. Therefore, statistics comparing RF and CB-2 in elderly patients with AF are scarce. In order to identify the approach with maximal benefits to the elderly patients, the costs and clinical outcomes were compared between CB-2 and RF for elderly patients in China in this study.

## Methods

### Patient selection

This retrospective cohort study compared consecutive patients, aged > 75 years with drug-resistant paroxysmal or short-lasting persistent AF, who underwent their first pulmonary vein isolation (PVI) using either the RF or CB-2 ablation technique in our center between September 2016 and April 2019. The choice of ablation technique was not random and was based on the discretion of the operating electrophysiologist. Paroxysmal AF (PAF) was defined as AF spontaneously occurring lasting > 30s and < 7 days. Short-lasting AF was defined as AF lasting from 7 days to 6 months before ablation [10]. Exclusion criteria were as follows: prior PVI, left atrium (LA) diameter > 50 mm, severe valvular heart disease, inability to provide informed consent, contraindications to post-interventional oral anticoagulation, or life expectancy < 1 year and patients with any additional ablation after PVI. Computed tomography (CT) scanning of the left atrium was employed to assess the PV anatomy. In addition, transesophageal echocardiography was performed prior to ablation in all patients to assess the LA diameter and exclude the intracavitary thrombi 24 h prior to the procedure. All patients signed informed consent for the ablation procedure.

### Periprocedural management

The ablation procedures were performed under conscious sedation and analgesia with appropriate doses of midazolam and alfentanil fentanyl. Next, the transseptal puncture was performed using fluoroscopic guidance by modified Brockenbrough technique. Transseptal puncture was followed by administration of an intravenous bolus of heparin (100 IU/kg) to maintain an activated clotting time of 300–350 s. Subsequently, selective PV angiographies were performed to identify the individual PV ostia. The esophagus was not monitored during the procedure. Administration of anti-arrhythmic drugs (AADs) was stopped 3 months after ablation. The choice of anticoagulant durs post-ablation was based on patient‘s wishes. Patients could choose warfarin or new oral anticoagulants for anticoagulation post-ablation. Hospitalization may be prolonged, because multiple international normalized ratio (INR) tests are necessary during hospitalization for patients received warfarin.

### Irrigated RF ablation

The 3-D reconstruction of the LA and PV ostia was performed using an electroanatomic mapping system (Carto 3, Biosense Webster Inc., USA). Two 8.5-Fr long sheaths (St. Jude Medical) were introduced into the LA. A circumferential mapping catheter (Lassow, Biosense Webster) was introduced into the LA through long sheath to assess the geometrical conformation. Subsequently, RF was applied using an open-irrigated tip catheter (Thermocool, Biosense Webster; Navistar Thermocool SmartTouch, Biosense Webster) in a power-controlled mode with a threshold of 35 W (flow rate, 17–20 mL/min) and maximum temperature of 48 °C. A 30 W power was limited to the posterior sites. For Thermocool SmartTouch™, contact force data were available to the operator throughout the procedure, to achieve at least 10 g (mean) with a vector perpendicular to the tissue and an upper limit of 50 g. No ablation-index (AI)-guided ablation was applied in these patients. The ablation strategy was performed around the PV ostia (creating contiguous focal lesions at a distance of > 5 mm from the ostia of the PVs resulting in circumferential lines) without additional adjunctive left atrial ablation. The endpoint of PVI was to obtain complete electrical isolation of PVs and confirmation of bidirectional block with a waiting time of 20 min after the final application. After isolation, if the AF did not convert to sinus rhythm (SR), external electrical cardioversion (ECv) was performed.

### Cryoballoon ablation

A 15-Fr steerable sheath (FlexCath, Medtronic, Minneapolis, MN, USA) was advanced through the transseptal puncture. Then, a second-generation 28 mm-cryoballoon (Arctic Front Advance, Medtronic) was introduced into the sheath, inflated, and advanced to the ostium of each PV. The PV occlusion was assessed by venous angiography. Optimal vessel occlusion was achieved when selective contrast injection showed total contrast retention without backflow to the atrium. After the occlusion was documented, ablation was performed with at least two applications per vein, each for 150–180 s. The PV activity was recorded using Circular Achieve Catheter (Achieve, Medtronic) at a proximal site in the ostium prior to ablation in each vein. During the ablation of right PVs, a quadripolar catheter was inserted in the superior vena cava to monitor phrenic nerve palsy (PNP) by pacing the right phrenic nerve with a 1500-ms cycle and a 20-mA output. The freezing cycle was terminated immediately after a loss of capture, or the strength of right hemidiaphragmatic contractions was attenuated. No any additional RF ablations were applied during or after cryoablation were included. The Achieve Catheter was reintroduced, and the bidirectional block was checked with a waiting time of 20 min after the last application. After isolation, if the AF did not convert to SR, external ECv was performed.

### Clinical follow-up

After discharge, the patients were scheduled for follow-up visits with baseline ECG and 24 h Holter monitoring at 3, 6, 12, 18, and 24 months. Additional telephonic interviews were conducted regularly. In the case of symptoms suggestive of recurrent arrhythmia, additional visits were recommended.

### Study endpoints

The primary endpoints were freedom from AF or atrial tachycardia at 12 and 24 months after the procedure. Atrial arrhythmias recrudescence was defined as any symptomatic or asymptomatic atrial arrhythmia lasting > 30 s after completing the blanking period after catheter ablation. Secondary endpoints were complications.

Early recurrence of atrial arrhythmia (ERAA) in the hospital was defined as any documented episode of AF or atrial tachycardia lasting for > 30s post-ablation during hospitalization [11–13]. Hospitalization costs refer to all the expenses incurred by the patient during hospitalization such as (1) diagnosis, (2) procedural equipment and disposable supplies, (3) procedure, (4) medication, and (5) comprehensive medical (care, bed, and dietary).

### Statistical analysis

Continuous measures are expressed as the mean ± standard deviation (SD) and compared using Student’s t-test. Categorical variables were compared using a chi-squared or Fisher’s exact test. A value of *P* < 0.05 was considered statistically significant. For Kaplan–Meier plot and patients at risk, a log-rank test was used to compare the AF recurrence-free survival between the groups. Statistical analysis was done using SPSS (version 22.0, SPSS Inc., Chicago, IL, USA) and GraphPad Prism 6 (GraphPad Software Inc., USA).

## Results

### Patient characteristics

A total of 324 elderly (> 75 years) patients with paroxysmal or short-lasting persistent AF were evaluated in this study (176 by RF and 148 by CB-2). About 216/324 (66.7%) patients presented PAF, and 108/324 (33.3%) patients presented short-standing persistent AF. The mean age of the cohort was 78.8 ± 2.4 years, and 123 (37.96%) of the patients were female. The mean CHA_2_DS_2_-VASc score was 3.09 ± 0.881, and HAS-BLED was 1.80 ± 0.94. The mean left ventricular ejection fraction (LVEF) was 57.7 ± 5.9%, and mean LA anteroposterior diameter was 42.6 ± 4.2 mm. Overall, no significant difference was detected in any baseline characteristic between the groups. However, the patients received warfarin after ablation differed between the two groups (31.8% vs. 49.4%, *P* = 0.002), and the remaining patients received new oral anticoagulation (NOAC) (Table 1).

**Table 1:** Patient characteristics

	CB-2 group (*n* = 148)	RF group (*n* = 176)	*P* value
Age, years	78.9 ± 2.32	78.8 ± 2.47	0.585
Male sex, *n* (%)	74 (50)	83 (47.2)	0.656
Short-lasting persistent AF, *n* (%)	51 (35.1)	57 (33)	0.68
BMI	23.0 ± 3.8	23.6 ± 4.	0.191
LVEF (%)	58.4 ± 5.8	57.1 ± 5.9	0.055
LA diameter, mm	42.1 ± 4.1	43.0 ± 4.2	0.053
CHA_2_DS_2_-VAS_C_ score	3.09 ± 0.79	3.07 ± 0.94	0.829
HAS-BLED score	1.83 ± 0.88	1.80 ± 1.00	0.777
Medical history, *n* (%)			
Coronary artery disease, *n* (%)	54 (36.5)	75 (42.6)	0.305
LV hypertrophy, *n* (%)	1 (0.7)	1 (0.6)	1.000
Chronic kidney disease, *n* (%)	4 (2.7)	6 (3.4)	0.759
Hypertension, *n* (%)	74 (50)	90(51.1)	0.911
Hyperlipidemia, *n* (%)	32 (21.6)	39 (22.2)	1.000
Diabetes, *n* (%)	25 (16.9)	38 (21.6)	0.325
Warfarin after ablation, *n* (%)	47 (31.8)	87(49.4)	0.002
New oral anticoagulants, *n* (%)	101(68.2)	89(50.6)	0.008
Contact-force catheter applied in the RF ablation, *n* (%)	–	48(27.3)	–

Data are expressed in mean ± standard deviation or number and percentage

CB-2, second-generation cryoballoon; RF, radiofrequency; BMI, body-mass index; LVEF, left ventricular ejection fraction; LA, left atrial; LV hypertrophy, left ventricular hypertrophy

### Procedural results

Procedural data are summarized in Table 2. Accordingly, the success of PVI was similar between CB-2 and RF (97.8% vs. 98.4%, *P* = 0.416). Statistically, compared to the RF group, the total procedure time and the left atrial dwell time (the length of time the catheter was present in the LA during the procedure) were shorter in the CB-2 group (112.9 ± 11.1 vs. 135.1 ± 9.9 min, *P* < 0.001; 53.7.4 ± 9.0 vs. 65.0 ± 9.0 min, *P* < 0.001). However, the fluoroscopy time was longer in the CB group than in the RF group (22.1 ± 3.3 vs. 18.5 ± 3.6 min, *P* < 0.001).

**Table 2:** Procedural results

	CB-2 group (*n* = 148)	RF group (*n* = 176)	*P* value
Pulmonary vein isolation rate (%)	97.8	98.4	0.416
Procedure time, min	112.9 ± 11.1	135.1 ± 9.9	< 0.001
The left atrial dwell time, min	53.7 ± 8.9	65.1.9 ± 9.0	< 0.001
Fluoroscopy time, min	22.1 ± 3.3	18.5 ± 3.6	< 0.001

Data are expressed in mean ± standard deviation or number and percentage

CB-2, second-generation cryoballoon; RF, radiofrequency

### Complications

Complications were presented in 16 patients with no significant differences between the two groups (CB-2 group, 5 (3.38%) and RF group, 11 (6.25%), *P* = 0.307). None of the patients died due to a procedure-related event during follow-up. Strikingly, groin hematoma occurred in five patients in the RF group and two patients in the CB-2 group. One patient in the RF group developed a femoral pseudoaneurysm that required thrombin injection; the other patients did not require further intervention. Stroke occurred in one patient in the CB-2 group. In this case, emergency cerebrovascular revascularization was successfully performed. Transient PNP was presented in one case only in the CB-2 group with complete resolution after 3 days. Pericardial tamponade occurred in one patient in the CB-2 group and two in the RF group. Furthermore, surgical treatment was essential only in the patient from the CB-2 group; the other patients of tamponades were successfully treated by pericardiocentesis. Two patients in the RF group showed mild pericardial effusions, but no invasive treatment was necessary. One patient in the RF group experienced cardiogenic shock after the procedure. In both groups, neither atrio-esophageal fistula nor PV stenosis (PVS) was reported. A summary of the complications is provided in Table 3.

**Table 3:** Clinical outcomes and costs

	CB-2 group (*n* = 148)	RF group (*n* = 176)	*P* value
ERAA, *n* (%)	21 (14.2)	38 (23.3)	0.047
Recurrent atrial arrhythmia, *n* (%)	35 (24.3)	50 (27.8)	0.375
Complications, *n* (%)	5 (3.3)	11(6.2)	0.307
Hematoma, *n* (%)	2 (1.4)	5 (2.8)	0.460
Stroke, *n* (%)	1 (0.6)	0	0.457
Phrenic nerve palsy, *n* (%)	1 (0.6)	0	0.275
Inguinal pseudoaneurysms, *n* (%)	0	1 (0.6)	1.000
Pericardial tamponade, *n* (%)	1	2 (1.1)	1.000
Pericardial effusion, *n* (%)	0	2 (1.1)	0.502
Shock, *n* (%)	0	1 (1.1)	1.000
The length of stay after ablation (days)	1.94 ± 1.1	2.53 ± 1.6	< 0.001
Costs (CNY)	91,132.6 ± 3723.5	81,149.4 ± 6824.1	< 0.001
Procedure equipment and disposable supplies costs	75,734.8 ± 1169.1	63,550.5 ± 5496.0	< 0.001
Procedure costs	6249.3 ± 318.0	7319.4 ± 355.3	< 0.001
Diagnostic costs	3341.4 ± 843.5	3459.7 ± 1201.8	0.315
Medication costs	4322.7 ± 773.3	4109.1 ± 1169.4	0.058
Comprehensive medical costs	2249.9 ± 423.2	2384.4 ± 557.0	0.014

Data are expressed in mean ± standard deviation or number and percentage

CB-2, second-generation cryoballoon; RF, radiofrequency; ERAA, early recurrence of atrial arrhythmia; CNY, China Yuan

### Clinical outcomes

**Fig. 1:**
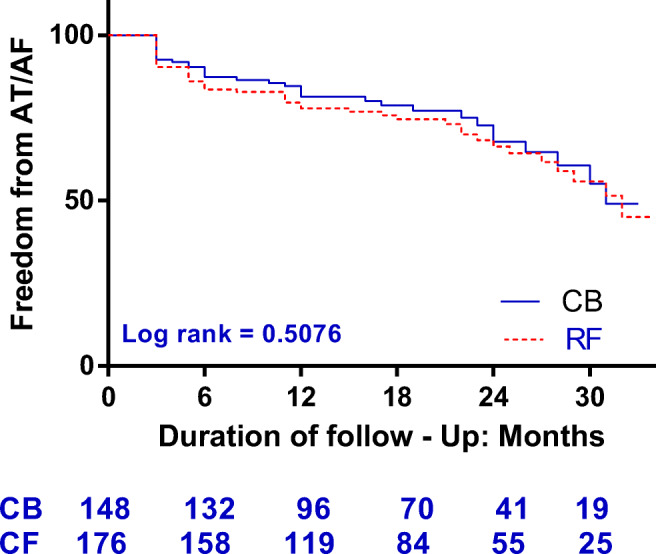
Kaplan-Meier survival curves of patients free from AF after the 3-month blanking period following the initial procedure

Until discharge, ERAA was less frequently observed in the CB-2 group than in the RF group (14.2% vs. 23.3%, *P* = 0.047). A total of 13 patients were lost to follow-up (CB-2 group, 5 patients, RF group, 7 patients). After an overall mean follow-up of 14.33 ± 9.07 (range: 3–34) months, the rate of recurrence of AF after a single procedure for CB-2 and RF was 24.3% (35/148 patients) and 27.8% (50/176 patients), respectively, and this difference did not reach statistical significance (*P* = 0.375). At the 12-month follow-up, 83.8% of the patients in the CB-2 group and 80.1% of the patients in the RF group (*P* = 0.47) and, at 24 months, 79.1% of the patients in the CB-2 group and 75.6% of the patients in the RF group (*P* = 0.507) remained free from any atrial arrhythmia recurrence as assessed by Kaplan–Meier method (Fig. 1).

### Costs and hospital stay

All costs were calculated in the year 2019 in Chinese Yuan (CNY) currency. The hospitalization costs were significantly higher in the CB-2 group than in the RF group (91,132.6 ± 3723.5 vs. 81,149.4 ± 6824.1CNY, *P* < 0.001). For further sub-analysis, the costs of procedural equipment and disposable supplies in the CB-2 group were higher than those in the RF group (75,734.8 vs. 63,550.5, *P* < 0.001); however, the procedural and comprehensive medical costs were higher in the RF group than in the CB group (6249.3 vs. 7319.4, *P* < 0.001; 2249.9 vs. 2384.4, *P* = 0.014). No significant difference was detected between the two groups with respect to the diagnostic and medication costs. These costs are summarized in Table 3. The mean length of hospital stay after ablation was significantly longer for the RF group (2.53 days) than the CB-2 group (1.94 days) (*P* < 0.001). There were six patients in the RF group whose length of stay after ablation exceeded 5 days: four due to pericardial tamponade/effusion, one due to cardiogenic shock, and one due to inguinal pseudoaneurysms. There were three patients whose length of stay after ablation exceeded 5 days in CB group: one due to PNP, one due to stroke, and one due to pericardial tamponade (Table 3).

## Discussion

Several clinical trials demonstrated that CA is safe and effective in elderly patients with AF. However, comparing about costs and clinical outcomes between different ablation energy in these patents is limited. The major findings of the current study are as follows:

1. The CB-2 group is associated with short procedure time, left atrial dwell time, and prolonged fluoroscopy time. Moreover, the rate of PVI was similar between the two groups.
2. ERAA is less frequent in the CB-2 group than the RF group. However, no differences were detected in terms of long-term success rates between RF and CB-2 groups.
3. The complication rates were similar between the CB-2 and RF groups.
4. The CB-2 group is associated with higher hospitalization costs but the shorter length of stay after ablation as compared to the RF group.

### Procedural characteristics

In the current study, procedure time and left atrial dwell time were shorter in the CB group than in the RF group, whereas fluoroscopy time was longer, which was consistent with the previous studies [7, 9, 14, 15]. Single transseptal puncture and single-step circumferential ablations with large cooling surface area might be the key factors to the short duration of the CB-2 group [9]. The prolonged fluoroscopy time in the CB-2 group might be attributed to the necessity of high-resolution fluoroscopy images to prove the balloon’s occlusion. However, in the RF group, catheter guidance could be achieved with the use of an electroanatomical mapping system, which leads to a short fluoroscopy time [7].

### Efficacy

Several trials of PVI concluded that the efficacy profile for older patients was comparable to that of the younger patients with a high rate of PVI. The results in the current study are in line with the previously published data, without significant difference in terms of PVI between the RF and CB groups [2, 4, 6, 7].

ERAA following ablation of AF is relatively common. Several studies reported that the variable rate of overall ERAA was 37.8%, (range: 16–67%), irrespective of the ablation energy applied [12, 16–19], with the incidence of ERAA being highest in the immediate post-ablation period (first 2 weeks) and decreasing progressively thereafter [17, 18]. Although ERAA might not imply long-term failure, the rates of late recurrences (LR) remain higher in patients with ERAA than in those without (54% vs. 7%) [19]. In the present study, ERAA occurred in 21 patients (14.2%) in the CB-2 group and 41 patients (23.3%) in the RF group with significant difference (*P* = 0.047). The mechanism ERAA was highly influenced by a post-procedural inflammatory reaction of the atrial tissue, maturation of ablation lesions, or imbalance of the autonomic system. Acute thermal injury effectuated by RF is characterized by coagulation and tissue necrosis, followed by a marked inflammatory response within the atria, resulting in cellular dysfunction and enhanced arrhythmogenicity. Conversely, CB results in the creation of dense, well-demarcated homogeneous lesions through a directed freezing process [20–22]. Thus, the relatively reduced inflammatory reaction explains a low rate of ERAA in the CB-2 group [11–13, 23].

As individuals aged, myocardium becomes increasingly infiltrated with fatty deposits and fibrosis. The elderly patients would be less likely to respond to AF ablation due to an altered electroanatomical atrial substrate [24]. However, Corrado et al. reported that 127 (73%) octogenarians with AF maintained sinus rhythm with a single procedure at 20 ± 14 months of follow-up [24]. In addition, the study by Kuck et al. demonstrated a similar rate of clinical success in patients with PAF after an RF or CB ablation [9]; however, patients > 75 years old were excluded from this trial. Recently, Ikenouchi et al. conducted a propensity-matched comparison of CB and RF for AF in elderly patients; the efficacy of PVI with CB was similar to that of RF [25]; however, the long-term clinical data are yet scarce. In the current study, the success rate was 83.8% in the CB group and 80.1% in the RF group without significant difference at 12 months post-procedure. At 24 months follow-up, 79.1% in the CB-2 group and 75.6% in the RF group were free from atrial arrhythmia (*P* = 0.507). Kaplan–Meier estimation did not reveal any significant difference in clinical outcomes after a single procedure between CB-2 and RF at the mean follow-up of 14.33 ± 9.07 months (log rank = 0.508), which was in line with the previously published data [25]. Therefore, elderly patients with AF could achieve acute and long-term success from catheter ablation by both CB-2 and RF.

### Safety

Data on the complications of elderly patients undergoing ablation of AF are yet limited and inconsistent. However, several trials investigating the complication rates in elderly patients undergoing ablation have shown favorable outcomes as compared to those in younger patients [2, 5, 6]. Thus, PVI did not increase the risk of complications in elderly patients. In the current study, the mean age of the cohort was 78.84 ± 2.40 years, and the incidence of complications was 4.93% without any significant difference between the CB-2 and RF groups (3.38% vs. 6.25%, *P* = 0.307). Taken together, these results showed that ablation is a safe procedure in elderly patients without an increased rate of complications, irrespective of CB or RF, which was similar to that reported previously [4, 25–27].

Furthermore, data on catheter ablation of AF stated that 0.3–1.3% patients of the CB ablation and 1.3–1.9% patients of the RF ablation suffered cardiac tamponade. In the current study, three patients experienced pericardial tamponade (0.92%): one patient in the CB-2 group and two patients in the RF group. The patient in the CB-2 group developed pericardial tamponade due to the perforation of the LA when we applied RSPV cryoablation, followed by surgical treatment. In the RF group, two patients fully recovered immediately after pericardiocentesis during the procedure. Mild pericardial effusion occurred in two patients in the RF group and recovered fully without extra-invasive treatment.

One patient in the CB-2 group developed hypotension and acute stroke at 2 h post-ablation. In this case, cerebral angiography was applied, which showed a thrombus in the M2 segment of the left middle cerebral artery. Emergency cerebrovascular revascularization was successfully performed immediately. In order to intravenous sedation during the procedure, fasting is necessary periprocedure. A prolonged peri- and post-procedural fasting resulted in low perfusion, which might be a major factor of acute stroke. Thus, for the elderly ablation patients, periprocedural fasting is essential, albeit perfusion should be under intensive focus.

The cardiogenic shock occurred in one patient in the RF group with severely reduced ejection fraction (40%) after AF ablation. Although AlTurki et al. found that CA for younger patients with AF and heart failure with reduced ejection fraction (HFrEF) was associated with a significant reduction in mortality and heart failure-related hospitalizations as well as an improvement in LVEF [28]. However, in elderly patients, HFrEF might increase the risk of cardiac adverse events during and post-ablation. These phenomena might be attributed to the further deterioration of heart function caused by intravenous sedation and surgical strike, which needs further substantiation. Overall, these findings showed that there was no increased risk of complications in the elderly population with both CB-2 and RF ablation. However, this finding needs to be confirmed in additional studies.

### Costs and hospital stay

Presently, data comparing the costs and the length of stay between RF and CB ablation for AF are sparse, especially for elderly patients. Yokokawa et al. analyzed 146 patients who had received ablation and demonstrated that CB has a higher cost than RF. The study found that the high cost in the CB group was mainly due to equipment cost. In addition, procedure duration significantly enhanced the cost as it is time-dependent cost based on the elements such as anesthesia services, use of the electrophysiology laboratory, and post-anesthesia recovery units [29]. In the current study, hospitalization costs are composed of five parts: diagnostic costs, procedure equipment and disposable supplies costs, procedure costs, medication costs, and comprehensive medical costs. The hospitalization costs were significantly higher in the CB-2 group than the RF group, which was primarily due to the high costs of procedure equipment and disposable supplies in CB-2 group (75,734.79 vs. 63,660.52, *P* < 0.001). However, double transseptal procedure and prolonged hospital stay after ablation led to the higher procedure and comprehensive medical costs in the RF group as compared to the CB group. Intriguingly, no significant different was observed in the diagnostic and medication costs between two groups.

In the case of elderly patients, CB-2 seemed to shorten the length of hospital stay after ablation due to the low rate of complications; combining complications would affect the length of stay after ablation severely. The other reason might be that the large subset of patients in the RF group received warfarin for anticoagulation post-ablation. Such patients would need additional hospital stay to monitor the oscillation of international normalized ratio (INR) and adjust the dose of the warfarin.

### Limitations

The present study has some limitations. First, this is a single-center retrospective study, and the choice of ablation technique was discrete by operating electrophysiologist, which could introduce selection bias. Future randomized studies with a larger population are essential. Second, asymptomatic episodes of atrial tachyarrhythmia might have been missed, and hence, the success rates might be overestimated. Persistent monitoring (such as CIED) is useful for long-term follow-up. Lastly, we did not systematically monitor the putative, mild, or asymptomatic complications (such as PV stenosis); therefore, the complication rate might have been underestimated.

## Conclusions

The results of the current study showed that both CB-2 and RF ablation are safe and effective to AF in elderly patients. CB-2 exhibited short procedure time, left atrial dwell time, and length of stay after ablation, as well as a low rate of ERAA. However, the costs and fluoroscopy time of CB-2 group are greater than that of the RF group. Additionally, lacking of AI guiding ablation is applied in the RF group, which showed a similar long-term success rate and complication in comparison with the CB-2 group. Thus, a strategy combining AI technology for AF ablation in elderly patients might be an optimal choice of approach.

## Data Availability

The datasets generated during and/or analyzed during the current study are available.
